# Crystallization and preliminary diffraction analysis of Wzi, a member of the capsule export and assembly pathway in *Escherichia coli*

**DOI:** 10.1107/S1744309110040546

**Published:** 2010-11-26

**Authors:** Simon R. Bushell, Hubing Lou, Gregor D. Wallat, Konstantinos Beis, Chris Whitfield, James H. Naismith

**Affiliations:** a Biomedical Sciences Research Complex, University of St Andrews, North Haugh, St Andrews KY16 9ST, Scotland; b Department of Molecular and Cell Biology, University of Guelph, Ontario N1G 2W1, Canada

**Keywords:** Wzi, polysaccharide capsules, *Escherichia coli*

## Abstract

External polysaccharide capsules provide a physical barrier that is employed by many species of bacteria for the purposes of host evasion and persistence. Wzi is a 53 kDa outer membrane β-barrel protein that is thought to play a role in the attachment of group 1 capsular polysaccharides to the cell surface. The purification and crystallization of an *Escherichia coli* homologue of Wzi is reported and diffraction data from native and selenomethionine-incorporated protein crystals are presented. Crystals of C-terminally His_6_-tagged Wzi diffracted to 2.8 Å resolution. Data processing showed that the crystals belonged to the orthorhombic space group *C*222, with unit-cell parameters *a* = 128.8, *b* = 152.8, *c* = 94.4 Å, α = β = γ = 90°. A His-tagged selenomethionine-containing variant of Wzi has also been crystallized in the same space group and diffraction data have been recorded to 3.8 Å resolution. Data processing shows that the variant crystal has similar unit-cell parameters to the native crystal.

## Abstract 2

Wzi is a membrane protein from *E. coli* thought to be involved in the attachment of capsular polysaccharides to the bacterial surface. This reports describes recombinant Wzi’s purification, crystallization and the results of initial diffraction studies.

## 1. Introduction

**Figure 1:**
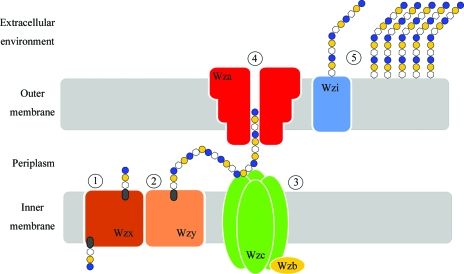
The Wzy-dependent capsule-assembly pathway in *E. coli*. Undecaprenol-linked CPS repeat units are synthesized by enzymes located in the cytoplasm and inner membrane and transported across the inner membrane *via* the flippase Wzx (1). These units are polymerized in concert with Wzy (2). Wzc is a tyrosine autokinase and Wzb is a cognate phosphatase (3). Phosphorylation and dephosphorylation are both required for high-level polymerization of the CPS. The periplasmic domain of the Wzc tetramer interacts with the base of Wza, the outer membrane channel required for translocation of CPS to the cell surface (4). Wzi is proposed to attach the newly translocated CPS to the outer membrane (5).

Extracellular polysaccharide capsules are produced by many species of bacteria to provide protection from the environment. In pathogens, capsules are often considered to be antiphagocytic structures, but they play other diverse roles depending on the species and the structure of the polysaccharide (Horwitz, 1982 ▶). The capsule primarily consists of long chains of repeat-unit polysaccharides that are firmly attached to the bacterial outer membrane by processes that are frequently not understood. Structural variation in *Escherichia coli* capsular polysaccharides (CPSs) gives rise to more than 80 different K (capsular) antigen types (Whitfield, 2006 ▶). Capsules are classified into four groups depending on various biochemical and genetic criteria (Whitfield, 2006 ▶; Whitfield & Roberts, 1999 ▶). The model system for group 1 capsules is the *E. coli* K30 serotype, which utilizes the Wzy-dependent pathway for capsular synthesis and export (Fig. 1 ▶).

In *E. coli* K30, the 16 kb capsule-biosynthesis (*cps*) gene cluster encodes activities required for the biosynthesis, export and assembly of the capsular structure. The locus contains 12 ORFs, four of which (*wzi*, *wza*, *wzc* and *wzb*) encode proteins involved in the assembly and export of CPS, while the remainder encode proteins responsible for CPS biogenesis. Undecaprenol diphosphate-linked CPS repeat units are synthesized at the cytoplasmic face of the inner membrane and are transported across the bilayer by the flippase Wzx. In the periplasm, these building blocks are polymerized by Wzy (Whitfield, 2006 ▶). Higher order polymerization is facilitated by Wzc, a transmembrane tyrosine kinase whose function is moderated by Wzb, its cytoplasmic cognate phosphatase (Hagelueken *et al.*, 2009 ▶; Wugeditsch *et al.*, 2001 ▶). The completed polymer is then transported to the bacterial surface *via* Wza, which acts as the pathway’s outer membrane channel (Beis *et al.*, 2004 ▶; Dong *et al.*, 2006 ▶; Nesper *et al.*, 2003 ▶).

Wzi is not essential for CPS synthesis (Drummelsmith & Whitfield, 1999 ▶); however, *wzi* knockouts in both *E. coli* and *Klebsiella* spp. (which use virtually identical Wzi, Wza, Wzb and Wzc proteins; Rahn *et al.*, 1999 ▶) show a profoundly altered morphology of the capsule (Alvarez *et al.*, 2000 ▶; Rahn *et al.*, 2003 ▶). This is the result of a markedly increased secretion of unattached exopolysaccharide into the environment and a commensurate decrease in attached CPS (Alvarez *et al.*, 2000 ▶; Rahn *et al.*, 2003 ▶). Wzi proteins are found in representatives from pathogens including *E. coli* and the genera *Acinetobacter*, *Klebsiella*, *Providencia* and *Serratia*, as well as free-living bacteria such as *Shewanella*. Interestingly, several other bacteria utilize similar Wzy-dependent pathways for the biosynthesis of predominantly secreted, rather than attached, exopolysaccharides. Although these bacteria contain homologues of Wza, Wzb and Wzc (which are presumably needed to make and export polysaccharide), they lack any obvious homologue of Wzi. Examples of these exo­polysaccharides include amylovoran and stewartan from the plant pathogens *Erwinia amylovora* (Bugert & Geider, 1995 ▶) and *E. stewartii* (Carlier *et al.*, 2009 ▶), respectively, and colanic acid from *Escherichia coli* (Stevenson *et al.*, 1996 ▶). Taken together, the collective evidence strongly suggests that Wzi plays a key role in the attachment of the capsule to the bacterial surface. By determining its structure, it may be possible to determine how it performs this vital role. In this report, we describe the purification and crystallization of recombinant Wzi and report on the progress of crystallographic diffraction studies.

## 2. Experimental

### 2.1. Expression and purification

DNA encoding the Wzi homologue from *E. coli* B44 (O9:K30:H^−^) was amplified using PCR primers that introduced a C-terminal hexahistidine tag into the gene product and was cloned into a pBAD24 vector, forming the plasmid pWQ192, as described pre­viously (Rahn *et al.*, 2003 ▶). N-terminal tagging was inappropriate as Wzi has a signal sequence that is cleaved during passage across the inner membrane. For the expression of native Wzi-His_6_, overnight cultures of *E. coli* DH5α (transformed to ampicillin resistance with pWQ192) were grown at 310 K in Luria–Bertani (LB) medium supplemented with ampicillin (100 µg ml^−1^) and used to inoculate 10 l LB broth. Expression of Wzi was induced *via* the addition of 0.02%(*w*/*v*) arabinose upon the cells reaching an OD_600_ of ∼0.6. Expression continued for 4 h at 310 K before the cells were harvested by centrifugation and stored at 193 K until required.

For the expression of selenomethionine-variant Wzi-His_6_, the methionine auxotroph *E. coli* B834 (DE3) strain was transformed to ampicillin resistance with pWQ192. Cultures of the transformed bacteria were grown overnight in LB in the presence of 100 µg ml^ −1^ ampicillin. Overnight cultures were harvested by centrifugation (4250*g* for 20 min at 293 K) and washed twice with PBS before being used to inoculate 6 × 1 l Glucose-Free SelenoMet Medium (Molecular Dimensions, Newmarket, England) supplemented with 0.4%(*v*/*v*) glycerol as a carbon source and 100 µg ml^−1^ ampicillin. The media contained 40 µg ml^−1^ l(+)-selenomethionine and were prepared according to the manufacturer’s instructions. Cells were grown at 310 K to an OD_600_ of ∼0.6 and expression was induced by the addition of 0.02%(*w*/*v*) arabinose for 4 h at 310 K. Expression was continued overnight at 298 K before the cells were harvested by centrifugation at 7000*g* and frozen at 193 K until required.

Both native and selenomethionine-labelled recombinant Wzi-His_6_ were purified from harvested cells using a modified protocol adapted from Rahn *et al.* (2003 ▶). Frozen cells were thawed in buffer *A* (20 m*M* phosphate buffer pH 7.4, 50 m*M* NaCl) and mechanically lysed using a pre-chilled Constant Systems Z Plus series cell disrupter (Constant Systems, Northants, England). Unbroken cells and cell debris were removed by centrifugation at 6000*g* for 20 min. The membrane fraction was isolated from the resultant supernatant by ultracentrifugation at 120 000*g* for 1 h. Inner membranes in the total membrane fraction were selectively solubilized by resuspending the pellet in buffer *A* containing 2%(*w*/*v*) *N*-lauroylsarcosine (Sigma–Aldrich UK, catalogue No. L5125) and incubating at 277 K with stirring for 3 h. The volume of this solubilization was 20 ml per litre of original culture. The outer membrane fraction was isolated as a pellet after further ultracentrifugation at 120 000*g* for 1 h and solubilized overnight in 120 ml buffer *A* containing 0.5%(*w*/*v*) SB3.14 [3-(*N*,*N*-dimethylmyristylammonio)propanesulfonate; Sigma–Aldrich UK, catalogue No. T7763]. Insoluble material was removed by ultracentrifugation at 120 000*g* for 1 h. The supernatant was applied onto a 5 ml HisTrap nickel column (GE Healthcare, Uppsala, Sweden) pre-equilibrated in 20 m*M* sodium phosphate pH 7.4, 50 m*M* NaCl, 0.05%(*w*/*v*) SB3.14. Once Wzi-His_6_ was bound, the column was washed with five column volumes of 20 m*M* sodium phosphate pH 7.4, 50 m*M* NaCl, 0.1%(*w*/*v*) LDAO (*n*-dodecyl-*N*,*N*-dimethylamine-*N*-oxide; Anatrace, Maumee, USA, catalogue No. D360). Wzi-His_6_ was eluted by a step gradient of wash buffer with increasing amounts of imidazole (12.5, 25, 50, 100 and 500 m*M*). Wzi-His_6_ readily eluted in 50 m*M* imidazole, which is an unusually low concentration for a hexahistidine-tagged protein; however, SDS–PAGE analysis of peak fractions showed it to be nearly homogenous. Final purification was performed *via* gel-filtration chromatography using a Superdex 200 10/300 size-exclusion column (GE Healthcare, Uppsala, Sweden) equilibrated in 20 m*M* Tris pH 7.4, 0.1% LDAO. Gel filtration of the selenomethionine form of Wzi-His_6_ used the same buffer but supplemented with 0.5 m*M* tris(2-carboxyethyl)­phosphine (TCEP).

**Figure 2:**
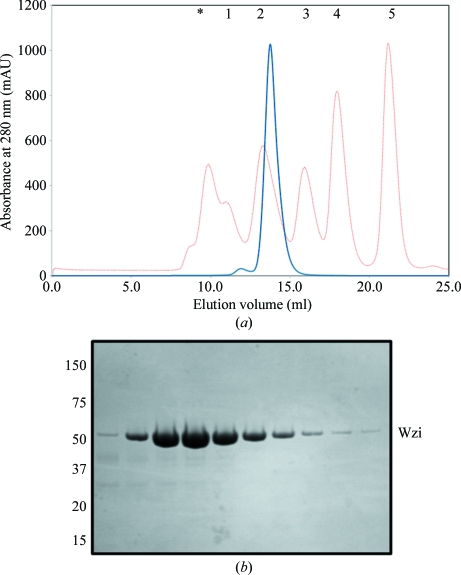
Purification of native Wzi-His_6_. (*a*) Elution profile of native Wzi-His_6_ (blue line) on size-exclusion chromatography using a Superdex 200 10/300 column. The elution was standardized using a mixture containing (1) thyroglobulin (670 kDa), (2) γ-­globulin (158 kDa), (3) ovalbumin (44 kDa), (4) myoglobin (17 kDa) and (5) vitamin B_12_ (1350 Da) (red line). The protein aggregate peak (marked with an asterisk) represents the void volume of the column. (*b*) SDS–PAGE analysis of peak fractions from SEC purification, showing the homogeneity of Wzi-His_6_ immediately prior to crystallization. The protein runs to its true size of 52 kDa upon boiling.

The elution profile indicated a single oligomeric state of Wzi-His_6_ in solution. Comparison with known protein standards suggested that Wzi elutes as a monomer–detergent complex (Fig. 2 ▶ *a*). When plotted on a calibration curve, the Wzi–detergent complex was calculated to have an approximate molecular weight of 80 kDa, which is in reasonable agreement with the molecular weight of Wzi of 53 kDa and an LDAO micelle size of ∼25 kDa (Strop & Brunger, 2005 ▶). The purification purity was monitored at all stages *via* SDS–PAGE (Fig. 2 ▶ *b*), with no other contaminants being visible on a Coomassie Blue-stained gel. The identity of recombinant Wzi-His_6_ was con­firmed by Western blot using an antihexahistidine monoclonal antibody and *via* MALDI–TOF mass-spectrometric analysis of peptides derived from a tryptic digest. The final yield of the native form of the protein was approximately 0.4 mg per litre of culture. The yield of the selenomethionine variant was higher at 0.7 mg per litre of culture, most likely owing to the longer overnight expression. Wzi was con­centrated to ∼12 mg ml^−1^ for crystallization trials.

### 2.2. Crystallization

**Figure 3:**
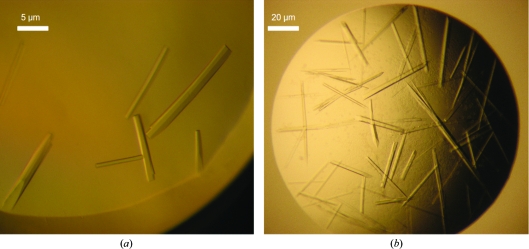
Crystals of native and selenomethionine-containing forms of Wzi-His_6_. (*a*) Native crystals grown in 0.03 *M* CaCl_2_, 25%(*v*/*v*) PEG 350 MME, 0.1 *M* MES pH 6.5. (*b*) Selenomethionine Wzi-His_6_ crystals grown in 0.15 *M* CaCl_2_, 23% PEG 350 MME, 0.1 *M* MES pH 6.0.

Several commercially available screens were employed to determine the crystallization space of Wzi-His_6_. Crystallization trays were generally constructed using a Cartesian Honeybee 963 liquid-handing robot in Innovadyne SD-2 96-well trays (IDEX Corp, Lake Forest, USA) with 60 µl precipitant dispensed into the reservoir. Crystallization was performed *via* sitting-drop vapour diffusion at 293 K by mixing 150 nl Wzi-His_6_ solution (at 12 mg ml^−1^) with 150 nl well solution. Native Wzi-His_6_ crystallized in several different conditions, with crystals taking between one and eight weeks to appear. Oval-shaped crystals grew in 12.5%(*w*/*v*) PEG 200 MME, 0.1 *M* sodium cacodylate pH 6.5; however, these crystals proved to be difficult to reproduce and were marked by severe anisotropy, most likely owing to their shape. The best crystals appeared in screens optimized for the crystallization of membrane proteins, specifically MembFac (Hampton Research, Aliso Vijeo, USA), MemGold and MemStart/MemSys (Molecular Dimensions, Newmarket, England). Broad screen hits were improved by varying the pH, salt and PEG concentrations. The most tractable crystals grew in 0.03 *M* CaCl_2_, 25%(*v*/*v*) PEG 350 MME, 0.1 *M* MES pH 6.5. The crystals resembled flattened hexagonal rods and usually grew attached to the bottom of the crystallization well. Small crystals appeared after ∼3 d and grew to approximately 30 µm in size within 7–10 d (Fig. 3 ▶ *a*).

Selenomethionine-labelled Wzi-His_6_ crystals grew under slightly different conditions, with the best crystals forming in 0.15 *M* CaCl_2_, 23% PEG 350 MME, 0.1 *M* MES pH 6.0. These crystals were of a similar morphology to those of the native form and grew to a comparable size (Fig. 3 ▶ *b*). However, these crystals grew more quickly, appearing overnight and growing to full size (30 µm) within 2–3 d.

All crystals used the drop itself as a cryoprotectant. Crystals were flash-cooled in liquid N_2_, either through immersion in pre-cooled robot pucks or by freezing the crystals directly in the cryostream prior to collection. Initial diffraction data showed that the majority of crystals frequently displayed moderate to severe anisotropy. Variation of cryoprotection conditions did not demonstrate any improvement of this problem. Rather, crystals were screened in advance in order to select the least anisotropic for further data collection.

### 2.3. Data collection

**Figure 4:**
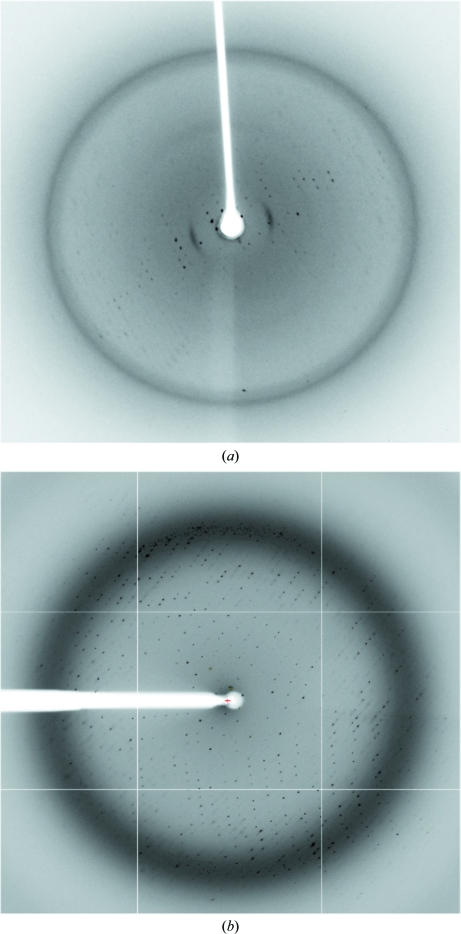
Native crystal diffraction. (*a*) Diffraction pattern from a typical native Wzi-His_6_ crystal showing high anisotropy. Data were collected in-house using a Saturn 944+ CCD detector attached to a Rigaku MicroMax-007 HFM X-ray source (λ = 1.54178 Å). Such crystals were discarded without further experimentation. (*b*) Diffraction pattern from the native Wzi-His_6_ crystal used to derive the data in Table 1 ▶.

Data sets from native Wzi-His_6_ crystals were collected on the ID14-4 beamline at the ESRF, Grenoble, France. A 2.8 Å resolution data set (Fig. 4 ▶) was collected from a single native Wzi-His_6_ crystal. The data were processed with *XDS* using the *xia*2 data-reduction pipeline (Kabsch, 2010 ▶; Winter, 2010 ▶). *xia*2 used *POINTLESS* and *LABELIT* to assist it in indexing the data (Collaborative Computational Project, Number 4, 1994 ▶; Sauter *et al.*, 2004 ▶). During processing, several wedges of data were reduced separately in *xia*2 and subsequently merged with *SCALA* (Evans, 2006 ▶).

Data processing showed that the native crystals belonged to the orthorhombic space group *C*222, with unit-cell parameters *a* = 128.8, *b* = 152.8, *c* = 94.4 Å, α = β = γ = 90°. The full details of this data set are described in Table 1 ▶. Matthews coefficient analysis suggested that either one or two monomers of the protein are present in the asymmetric unit (*V*_M_ = 4.46 or 2.23 Å^3^ Da^−1^, respectively), with a solvent content of 72 or 45%, respectively (Kantardjieff & Rupp, 2003 ▶; Matthews, 1968 ▶). Although 72% solvent content is at the upper range of commonly observed values, the anisotropic nature of the diffraction, the absence of any noncrystallographic symmetry and the fragility of the crystals point towards the presence of a monomer in the asymmetric unit.

Although the sequence of Wzi suggests that it is a β-barrel, we have not identified a matching structure and all attempts at molecular replacement failed. The selenomethionine variant of Wzi-His_6_ was crystallized in order to determine the phases using single-wavelength anomalous dispersion (SAD). Data sets from crystals that showed promising in-house diffraction were collected on beamline I24 at the Diamond Light Source (DLS), Didcot, England using a Pilatus 6M detector (λ = 0.9778 Å). Several data sets were collected, with the best example containing diffaction to 3.8 Å resolution. These data were indexed and scaled using *XDS* in *xia*2 using the same approach as described for the native form (Kabsch, 2010 ▶; Winter, 2010 ▶). Processing showed that these crystals also belonged to space group *C*222 and they showed similar unit-cell parameters to the native protein (Table 1 ▶). Fluorescence scans of crystals of the selenomethionine variant showed that selenomethionine had been incorporated into the protein. Data reduction indicated the presence of anomalous scattering by measuring the difference between |*I* ^−^| and |*I* ^+^|. We have not yet been able to solve the substructure of Se atoms.

## 3. Discussion

We have obtained crystals of the integral membrane *E. coli* protein Wzi. Although crystals appeared in initial screens, extensive optimization was required in order to obtain crystals which diffracted to beyond 6 Å resolution. The native crystals diffracted to 2.8 Å resolution, suggesting that it will be possible to report a well defined experimental structure for this protein. Sequence analysis suggests the protein will be a β-barrel, but with a structure different to those observed previously. Gel filtration and crystal packing are consistent with a monomeric protein. We anticipate that solution of the structure of the selenomethionine variant of Wzi-His_6_ will require higher quality diffraction data. Efforts to improve the crystal quality for the selenomethionine protein are continuing.

## Figures and Tables

**Table 1:** Data-collection and processing statistics Values in parentheses are for the last shell.

	Native Wzi	SeMet Wzi
Source	ESRF, ID14-4	DLS, I24
Wavelength (Å)	0.9255	0.9778
Oscillation (°)	0.65	1.0
Space group	*C*222	*C*222
Unit-cell parameters (Å, °)	*a* = 133.2, *b* = 152.3, *c* = 95.3, α = β = γ = 90	*a* = 128.8, *b* = 152.8, *c* = 94.4, α = β = γ = 90
Resolution (Å)	50–2.8	53–3.8
Total reflections	132346	41664
Unique reflections	23861	9484
Mosaicity (°)	0.72	0.14
Anomalous correlation	—	0.114 (0.047)
Completeness (%)	98.5 (98.7)	99.9 (100)
Multiplicity	5.5 (4.7)	4.4 (4.6)
Mean *I*/σ(*I*)	13.7 (2.7)	7.6 (3.0)
*R*_merge_ (%)	8.2 (52.2)	14.5 (40.3)
